# Supplementary material for: Dual-targeting CRISPR-CasRx reduces C9orf72 ALS/FTD sense and antisense repeat RNAs in vitro and in vivo
Source: Nat Commun. 2025 Jan 8;16:459. doi: 10.1038/s41467-024-55550-x (PMC11711508; doi:10.1038/s41467-024-55550-x)

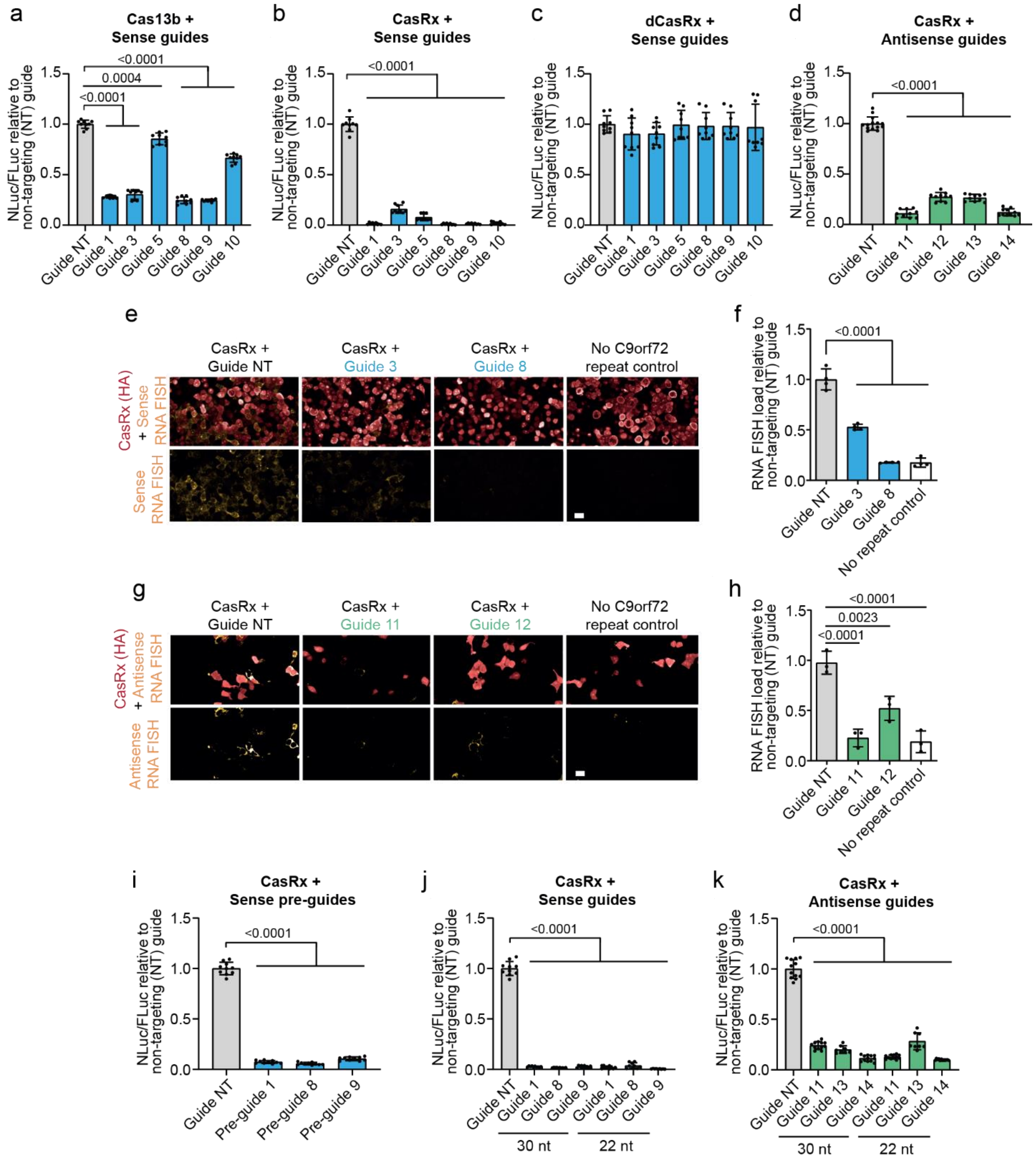

Supplementary Figure 1. CRISPR-CasRx can mature pre-gRNAs and reduce sense and antisense repeat-containing RNAs in a single construct with higher efficacy than Cas13b. (a-c) Sense NLuc reporter assays for **(a)** Cas13b, **(b)** CasRx, and **(c)** dCasRx using sense targeting gRNAs. **(d)** Antisense NLuc reporter signal following CasRx and antisense targeting gRNA application. In (a-d) each NLuc reading was normalised to FLuc for each well and further normalised to the non-targeting control gRNA. Data in (a-d) given as mean ± S.D, n=3 biological repeats (with technical replicates shown on graph), one-way ANOVA with Dunnett's test for post-hoc analysis, performed on the biological replicates. **(e)** Representative images of RNA-FISH for the sense G_4C_2 transcript and ICC for the HA tag of CasRx with different CasRx guides. Scale bars = 20 µm**. (f)** Quantification of sense RNA FISH load calculated as integrated intensity of nuclear RNA puncta per CasRx positive cell. Data given as mean ± S.D, n=4 biological repeats, one-way ANOVA with Dunnett's test for post-hoc analysis. **(g)** Representative images of RNA-FISH for the antisense C_4G_2 transcript and ICC for the HA tag of CasRx with different CasRx guides. Scale bars = 20 µm. **(h)** Quantification of antisense RNA FISH load calculated as integrated intensity of nuclear RNA puncta per CasRx positive cell. No repeat control indicates background signal of the LNA probe used for RNA FISH. Data given as mean ± S.D, n=3 biological repeats, one-way ANOVA with Dunnett's test for post-hoc analysis. **(i)** Sense targeting guides cloned into a pre-gRNA expressing plasmid and tested in our sense NLuc assay. **(j-k)** Testing of 30 nt and 22 nt gRNA variants of previously tested gRNAs in both the **(j)** sense and **(k)** antisense NLuc reporter assays. Data in (i-k) given as mean ± S.D, n=3 biological repeats (with technical replicates shown on graph), one-way ANOVA with Dunnett's test for post-hoc analysis, performed on the biological replicates. Source data are provided as a Source Data file.

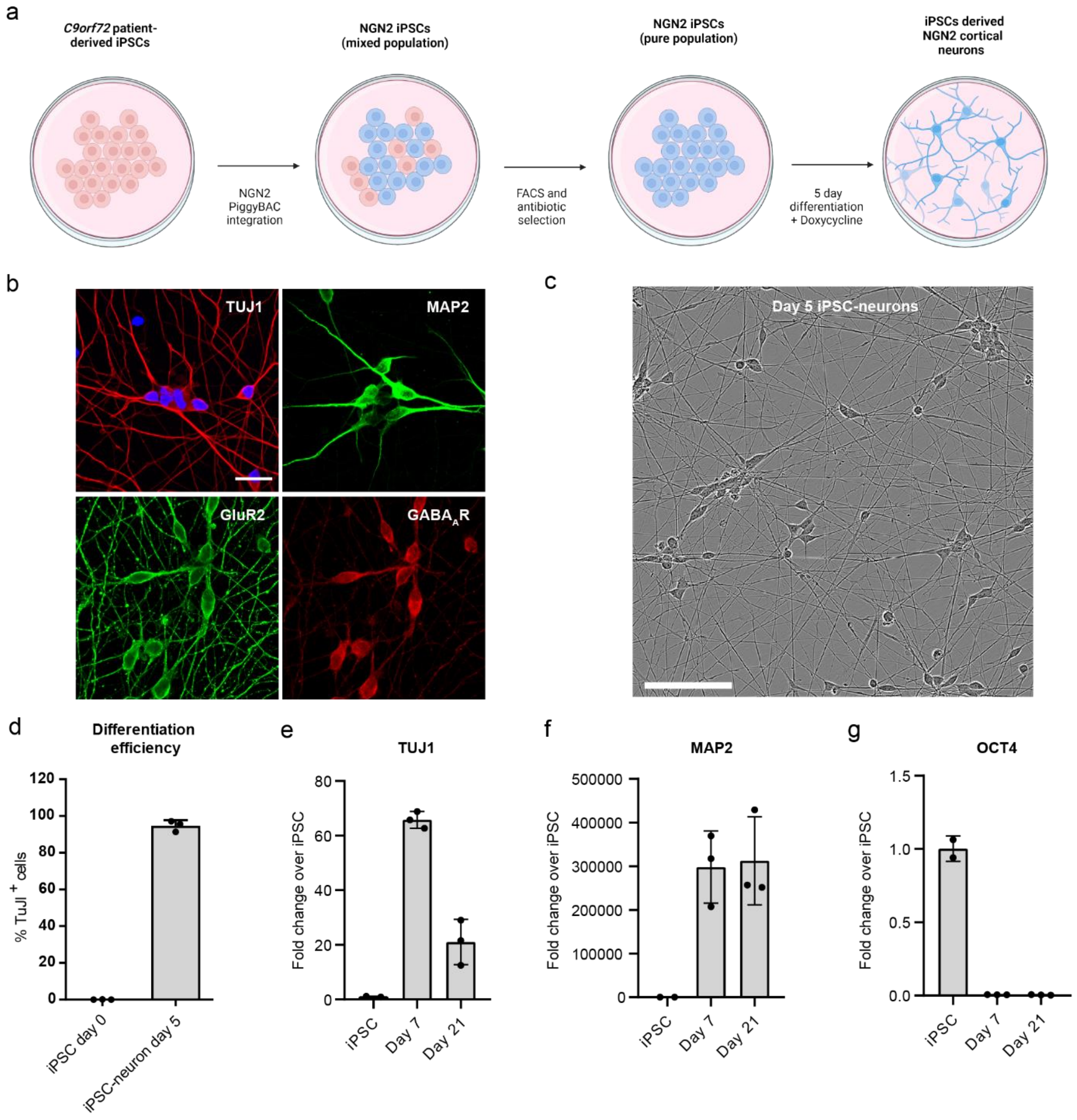

Supplementary Figure 2. Characterisation of NGN2 iPSC-neuron model. (a) Schematic NGN2 iPSC generation and i[3]Neuron differentiation protocol. A doxycycline-inducible NGN2 + BFP cassette was genomically inserted into *C9orf72* patient-derived iPSCs with piggyBAC transposase. iPSCs were selected for presence of the cassette and then rapidly differentiated into mixed cortical-like neurons via the i[3]Neuron protocol. Created in BioRender. Cammack, A. (2024) https://BioRender.com/f01i172. **(b)** Immunocytochemistry of differentiated NGN2 neurons after 5 days *in vitro* (DIV5) showing expression of neuronal markers $GABA_AR$, GluR2, MAP2 and TUJ1; scale bar = 50 µm. **(c)** DIV5 NGN2 neurons morphologically resemble neurons with neuronal projections visible by light microscopy; scale bar = 200 µm. **(d)** Quantification of % of TUJ1 positive cells at DIV5. Data presented as fold change compared to undifferentiated NGN2 iPSCs across n=3 technical replicates in a single induction of C9 line 1. **(e-g)** RT-qPCR analysis on RNA from DIV7 and DIV21 differentiated neurons show expression of **(e)** TUJ1, **(f)** MAP2, and loss of expression of pluripotency marker **(g)** OCT4. Data presented as fold change compared to undifferentiated NGN2 iPSCs across n=3 separate neuronal inductions of C9 line 1. Source data are provided as a Source Data file.

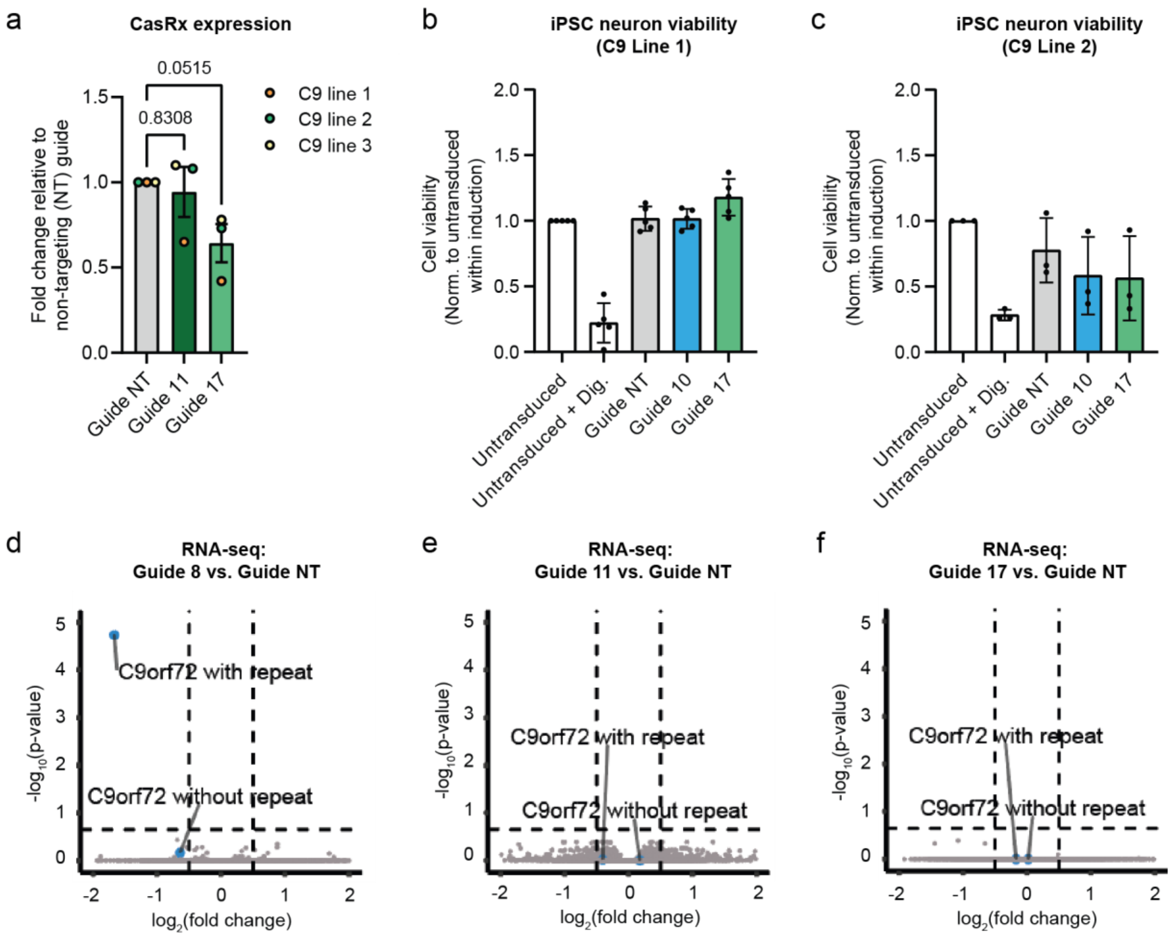

Supplementary Figure 3. CRISPR-CasRx expression in iPSC-neurons and viability/off target analysis. (a) RT-qPCR for CasRx expression in C9 lines transduced with lentivirus expressing CasRx and either non-targeting (NT) guide or antisense targeting guides 11 or 17. Data given as mean ± S.D, n=3 biological replicates (i.e. individual C9 lines), each shown as different colour data points (orange, green, and yellow). Two-way ANOVA with Dunnet's multiple comparisons test for post-hoc analysis**. (b-c)** Cell viability of neurons treated with CRISPR-CasRx lentivirus (with sense targeting guide 10 or antisense targeting guide 17) assessed 5 days post-transduction (DIV5) via CellTiter-Fluor^{TM} assay. Untransduced samples were included as controls, alongside the lytic reagent digitonin (dig), the positive assay control. Data given as mean ± S.D, n=5 biological replicates of C9 line 1, n=3 biological replicates of C9 line 2, one-way ANOVA with Holm-Sidak post-hoc analysis. **(d)** Volcano plots of DESeq2 analysis showing DEGs between neurons treated with CasRx lentiviruses expressing sense-targeting guide 8 compared to CasRx lentivirus expressing non-targeting (NT) guide with *C9orf72* transcripts grouped by those that contain intron1 and the repeats, and those that do not. **(e-f)** Volcano plots of DESeq2 analysis showing no DEGs between cells treated with CasRx lentiviruses expressing antisense-targeting guides 11 **(e)** or 17 **(f)**, compared to CasRx non-targeting (NT) control lentivirus. Dotted lines indicate thresholds for fold change on x axis (|$\log_2$FoldChange|>0.5) and *p* value on y-axis (adjusted p<0.05). n=3 independent inductions of C9 line 1. Source data are provided as a Source Data file.

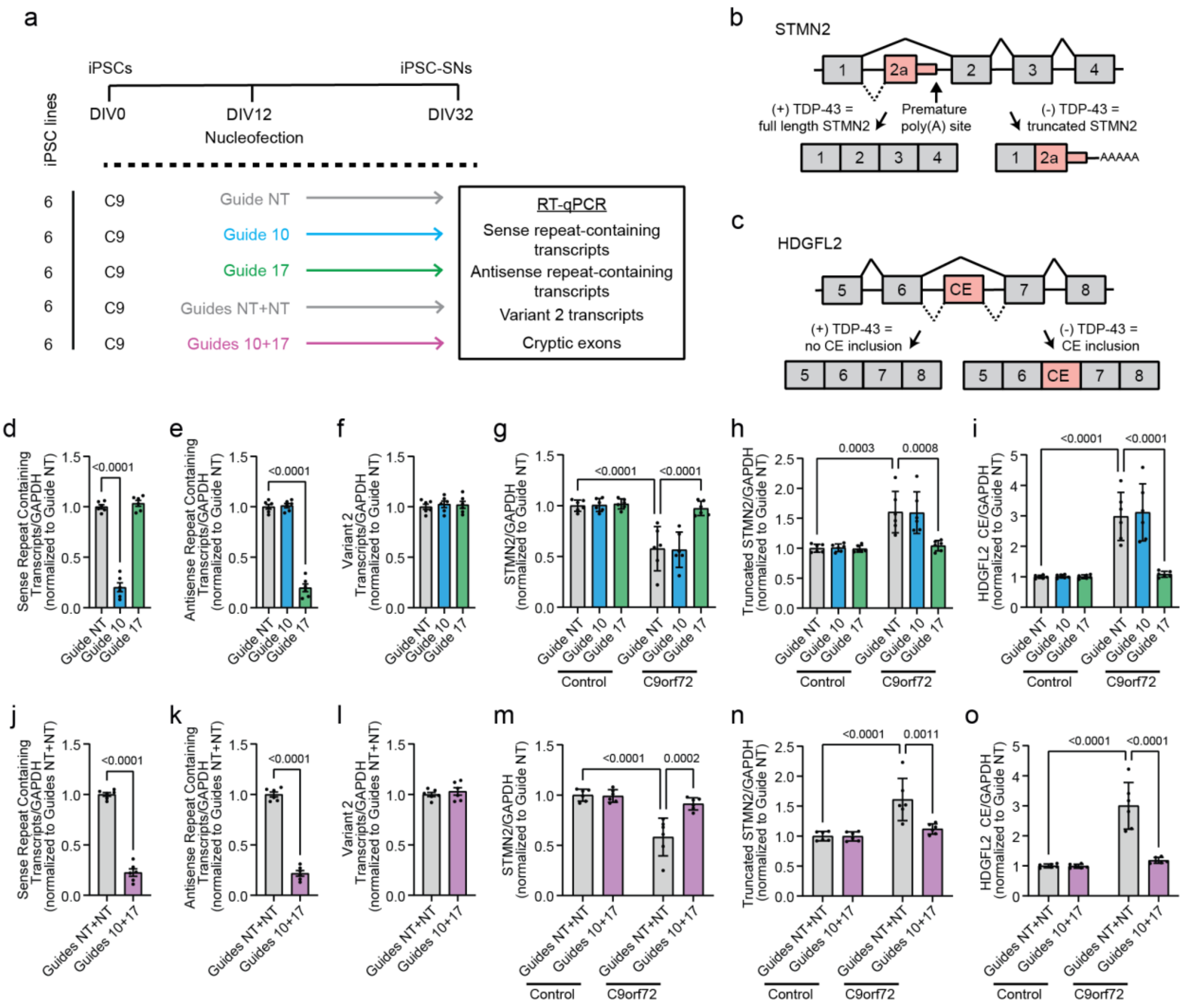

Supplementary Figure 4. CRISPR-CasRx expression in iPSC-spinal neurons prevents STMN2 and HDGFL2 cryptic exon inclusion. (a) Schematic of nucleofection paradigm for CRISPR-CasRx treatment in patient iPSC-derived spinal neurons (iPSNs). **(b-c)** Schematics of TDP-43-mediated splicing repression of cryptic exons in STMN2 and HDGFL2**. (d-f)** RT-qPCRs of sense, antisense, and variant 2 transcripts following CRISPR-CasRx treatment with single guides. **(g-i)** Antisense (guide 17) CRISPR-CasRx, but not sense (guide 10) CRISPR-CasRx, treatment significantly rescues cryptic exon expression in STMN2 and HDGFL2 and restores wild-type STMN2 expression. **(j-l)** RT-qPCRs of sense, antisense, and variant 2 transcripts following CRISPR-CasRx treatment with dual guides. **(m-o)** Dual guide CRISPR-CasRx, treatment significantly rescues cryptic exon expression in STMN2 and HDGFL2 and restores wild-type STMN2 expression. Two-way ANOVA with Tukey's multiple comparison test was used to calculate statistical significance throughout figure and data are presented as mean ± S.D with n=6 lines per condition. Source data are provided as a Source Data file.

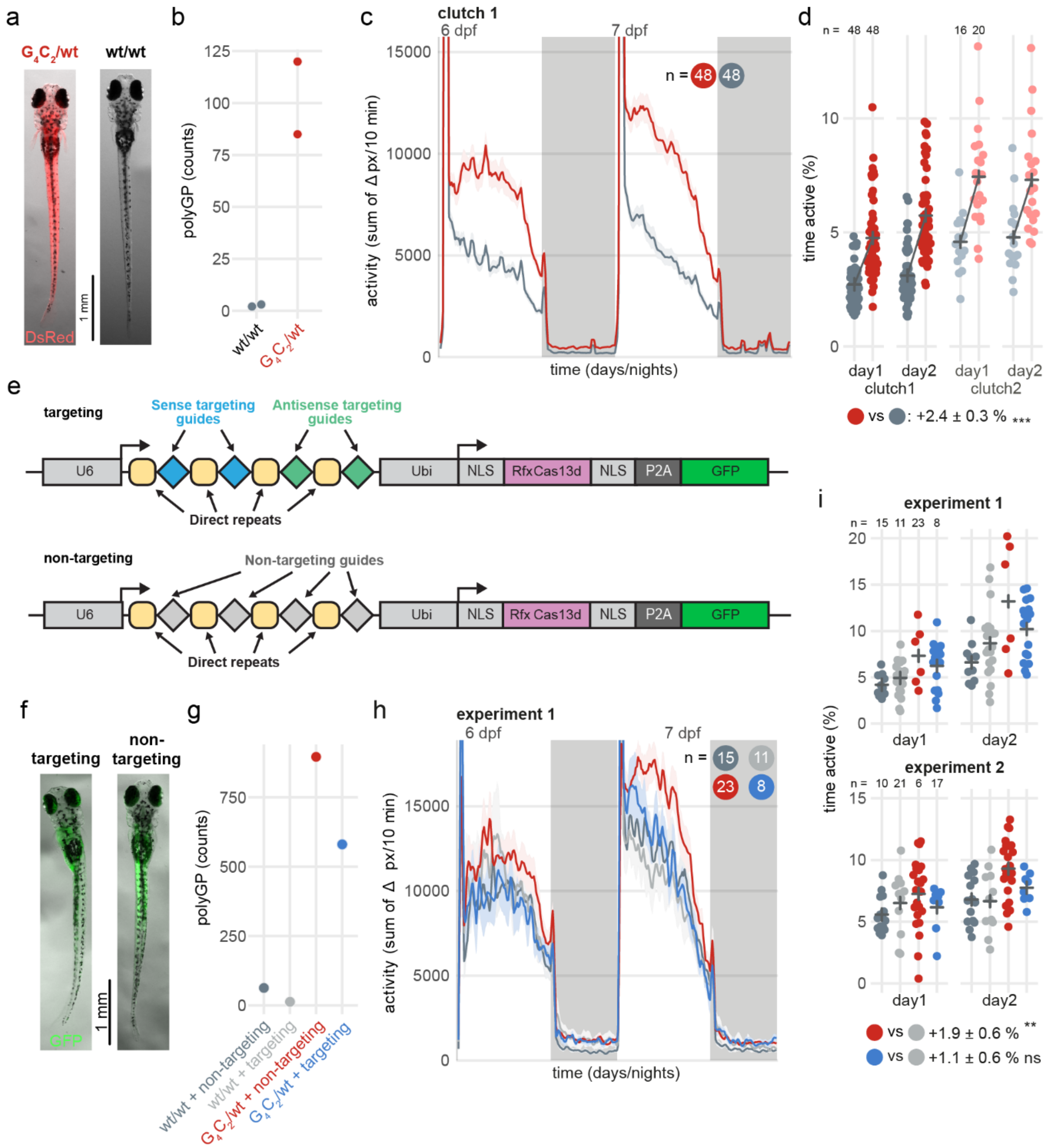

Supplementary Figure 5. CRISPR-CasRx rescues larval hyperactivity in a *C9orf72* zebrafish model. (a) An example *ubi:G4C2x45* heterozygous larva (G_4C_2/wt) expressing DsRed compared to a wild-type (wt/wt) sibling at 8 dpf. **(b)** Levels of polyGP DPRs in pools of 8-dpf larvae, measured by MSD. Each dot represents one clutch. **(c)** Activity (sum of Δ pixels/10 minutes) of wild-type (wt/wt, dark grey) and G_4C_2 heterozygous (G_4C_2/wt, red) larvae during 48 hr on a 14 hr:10 hr light:dark cycle (white background for days, grey background for nights). **(d)** Time spent active (% of each day) for each larva. Black crosses mark the group means. Compared to wild-type larvae (wt/wt, dark grey), *ubi:G4C2x45* heterozygous larvae (G_4C_2/wt, red) spent more time active during the day (***$p<0.001$). Statistics by likelihood-ratio test on a linear mixed effect model. **(e)** Schematic of the CRISPR-CasRx plasmid injected in *ubi:G4C2x45* heterozygous larvae and wild-type embryos. The flanking Tol2 arms are not shown. NLS, nuclear localisation signal; Ubi, ubiquitin promoter. **(f)** Example GFP imaging of 8-dpf larvae expressing CasRx with either targeting or non-targeting gRNAs. Targeting or non-targeting plasmid and Tol2 recombinase mRNA were co-injected at the single-cell stage. **(g)** Levels of polyGP DPRs in pools of 8-dpf larvae, measured by MSD. Each dot represents the mean of two technical replicates. **(h)** Activity (sum of Δ pixels/10 minutes) of wild-type (wt/wt) and *ubi:G4C2x45* heterozygous (G_4C_2/wt) larvae expressing CasRx and targeting or non-targeting gRNAs, as in (c). **(i)** Time spent active (% of each day) for each larva. As in (d), compared to control-injected wild-type larvae (wt/wt + non-targeting, dark grey), control-injected *ubi:G4C2x45* heterozygous larvae (G_4C_2/wt + non-targeting, red) spent more time active (**$p=0.01$). In contrast, the activity of *ubi:G4C2x45* heterozygous larvae expressing CasRx and targeting gRNAs (G_4C_2/wt + targeting, blue) was not significantly different to control-injected wild-type. Statistics by likelihood-ratio test on a linear mixed effect model and sample sizes are displayed in figure panels. Source data are provided as a Source Data file.

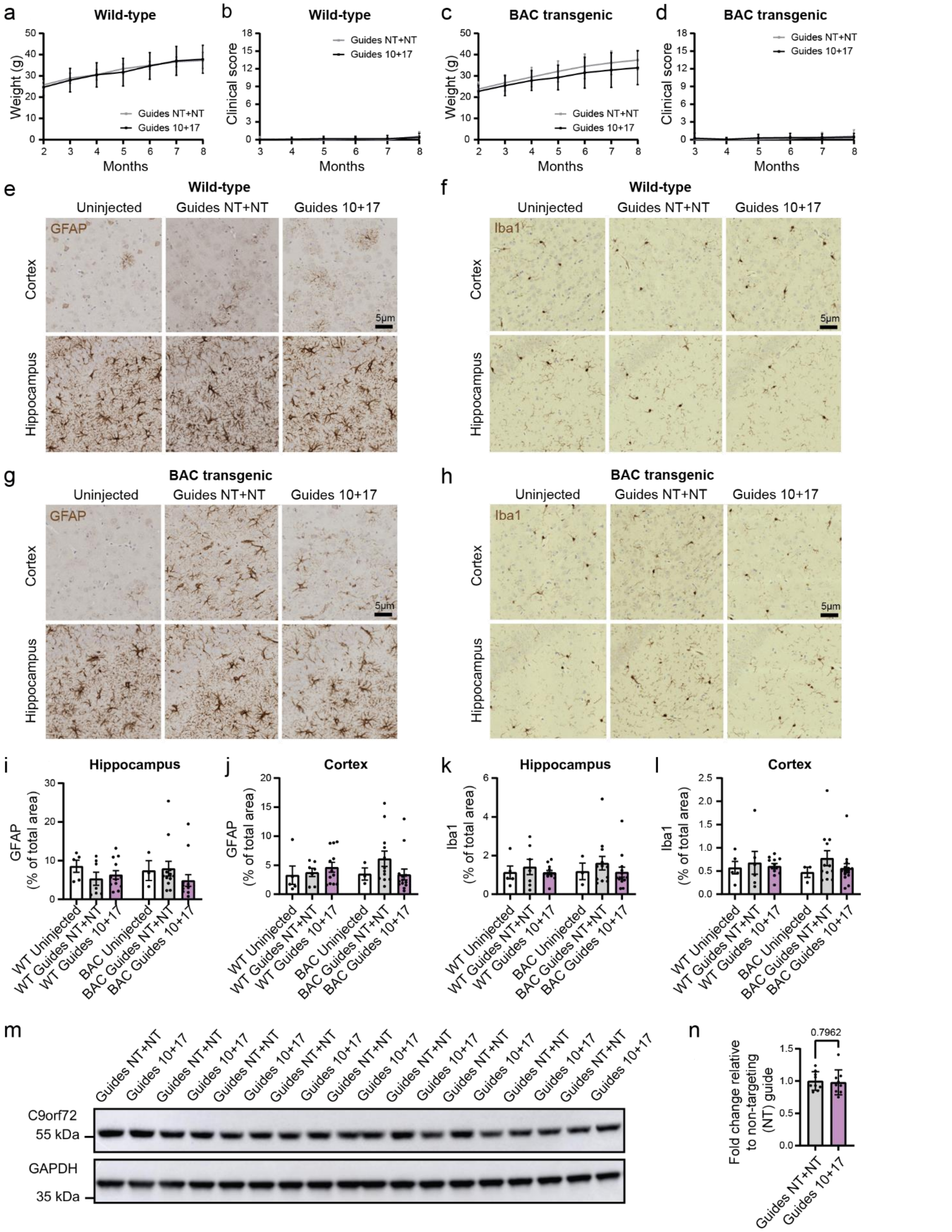

Supplementary Figure 6. CRISPR-CasRx treatment does not cause overt toxicity *in vivo* in wild-type or *C9orf72* BAC transgenic mice. (a-b) Monthly body weight analysis of **(a)** wild-type or **(b)** *C9orf72* BAC transgenic mice injected at P0 with either non-targeting (Guides NT+NT) or dual sense and antisense targeting (Guides 10+17) CasRx PHP.eB AAV from 2 months of age until end of the study. **(c-d)** Composite clinical score of **(c)** wild-type or **(d)** BAC transgenic mice injected at P0 with either non-targeting or Guide 10+17 CasRx PHP.eB AAV from 2 months of age until end of the study. Wild-type: n=12 Guide 10+17; n=7 Guide NT+NT. *C9orf72* BAC: n=14 Guide 10+17; n=12 Guide NT+NT. **(e-l)** GFAP and IbaI quantification in cortex and hippocampus of wild-type and BAC mice following AAV CRISPR-CasRx treatment. Wild-type: n=5 uninjected; n=12 Guide 10+17; n=6 Guide NT+NT. *C9orf72* BAC: n=3 uninjected; n=14 Guide 10+17; n=12 Guide NT+NT. One-way ANOVA used to calculate significance. **(m-n)** Western

blot quantification of C9orf72 protein in AAV CRISPR-CasRx-treated BAC mice. Band represents both mouse and human C9orf72 protein. Blot was run one time with all animals on the same membrane and is shown in figure. Uncropped blot is provided at the end of this Supplementary Information file. Two-sided unpaired Student's t-test used to calculate significance. n=9 per condition. Source data are provided as a Source Data file.

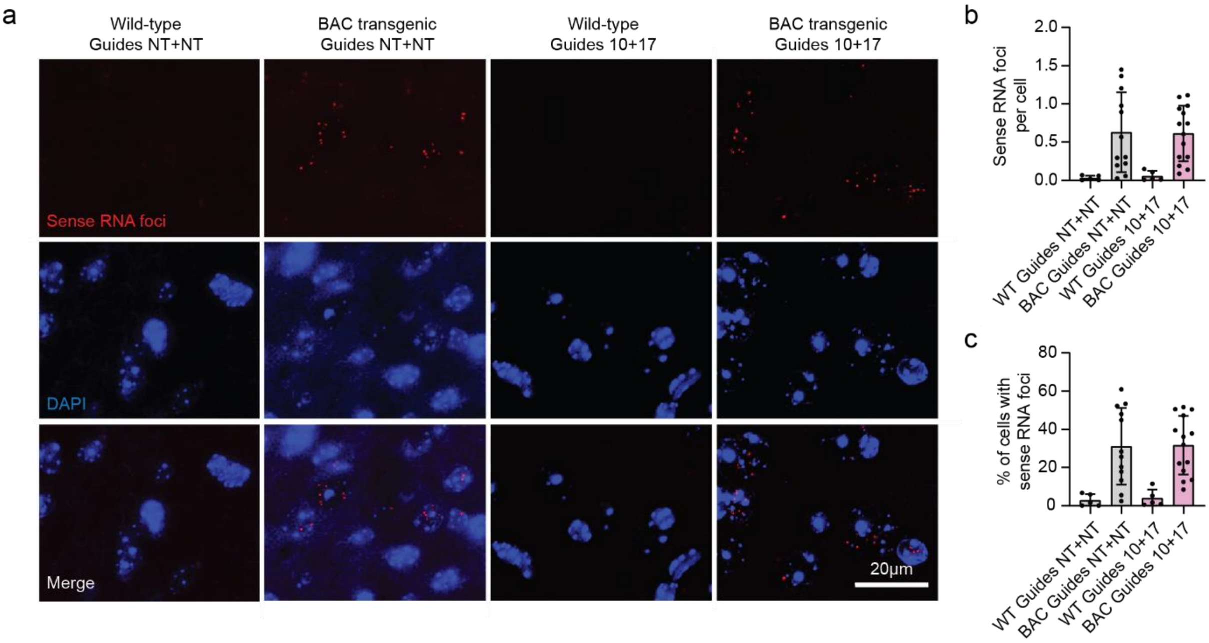

Supplementary Figure 7. CRISPR-CasRx treatment does not reduce RNA foci load in cortex of *C9orf72* BAC transgenic mice. (a-c) Representative images and quantifications of RNA foci load in BAC mice following AAV CRISPR-CasRx treatment. Scale bar represents 20 µm. Significance calculated with two-sided unpaired Student's t-test using BAC(+) animal groups only ($p>0.05$ not significant, two-sided unpaired t-test comparing BAC NT+NT to BAC 10+17). n=5 wild-type NT+NT; n=5 wild-type 10+17; n=12 BAC NT+NT; n=14 BAC 10+17. Source data are provided as a Source Data file.

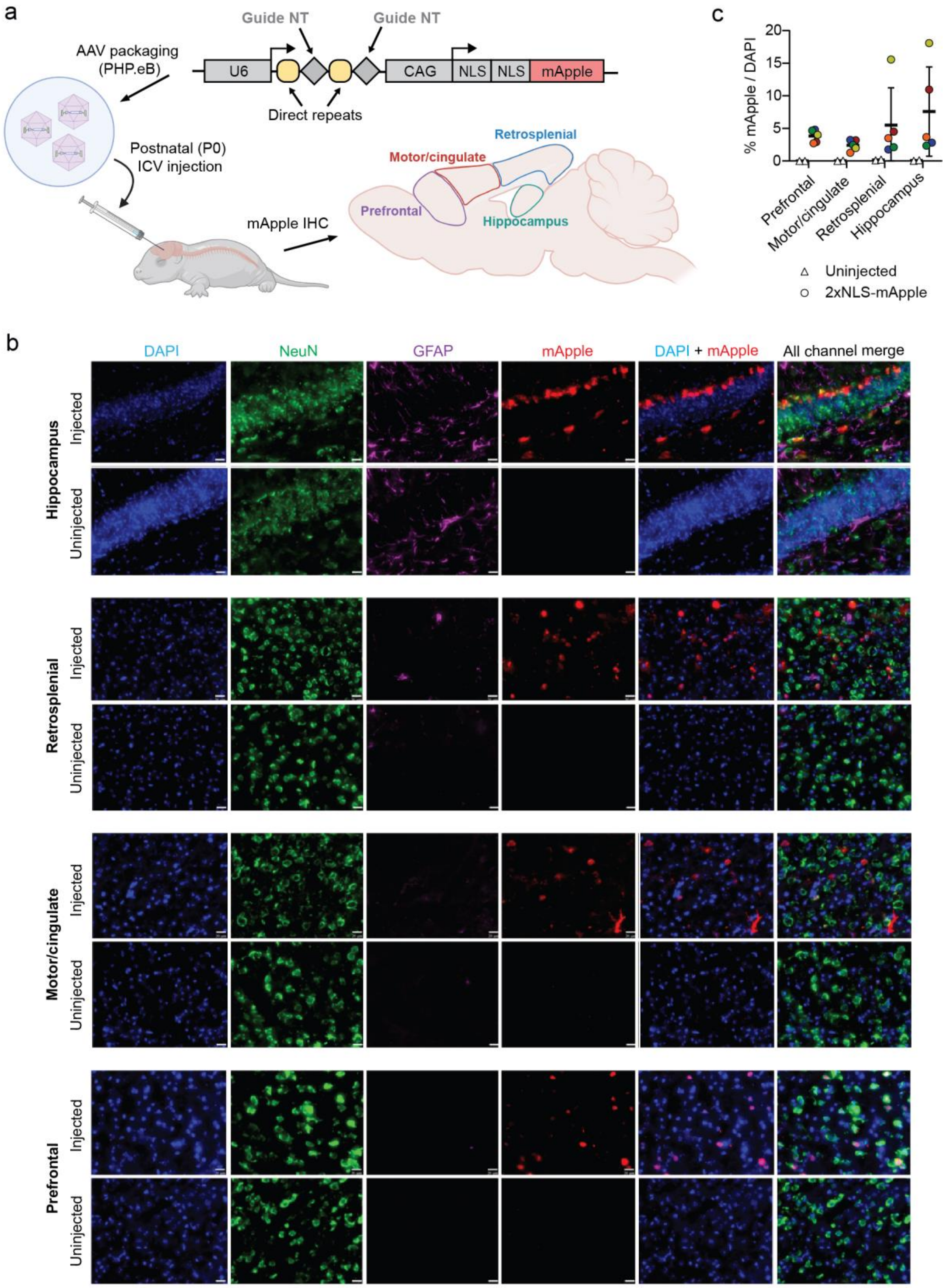

Supplementary Figure 8. 2xNLS-mApple reporter AAV displays low transduction efficiency in BAC mouse brain. **(a)** Schematic of mApple reporter AAV construct and study design in *C9orf72* BAC mice. Study was designed to match the plasmid vector, promoter (CAG), capsid (PHP.eB), titer (8E+9 vg per animal), and delivery method (P0 ICV) to the CRISPR-CasRx BAC mouse experiment. Brains were collected and mApple expression was quantified across the brain 4 weeks post-injection. Created in BioRender. Cammack, A. (2024) https://BioRender.com/u85i634 and https://BioRender.com/j31b211. **(b-c)** Representative images and quantifications (as % of DAPI positive cells) of mApple expression in various brain regions. All images were taken with equal settings and quantifications were done with equal thresholds. Each differently coloured dot in (c) represents the same animal across each brain region. Scale bar represents 20 µm. n=5 injected and n=2 uninjected animals. Source data are provided as a Source Data file.

iPSC line name	Common name	Sex (M/F)	Clinical diagnoses	Number of G_4C_2 repeats	Source of iPSCs
BS6	C9 line 1	F	C9orf72 ALS/FTD	~750	Chandran lab, Univ. of Edinburgh
DN19V4	C9 line 2	M	C9orf72 ALS	~638	Chandran lab, Univ. of Edinburgh
M211R2	C9 line 3	M	C9orf72 ALS	~960	Chandran lab, Univ. of Edinburgh

Supplementary Table 1. Demographic information for iPSC lines used in FACS experiments.

iPSC line name	Age at collection	Sex (M/F)	Clinical diagnosis	Source
CS0002	51	M	N/A	Cedars-Sinai
CS0201	56	F	N/A	Cedars-Sinai
CS9XH7	53	M	N/A	Cedars-Sinai
CS8PAA	58	F	N/A	Cedars-Sinai
CS1ATZ	60	M	N/A	Cedars-Sinai
CS2AE8	50	F	N/A	Cedars-Sinai
CS0NKC	52	F	C9orf72 ALS	Cedars-Sinai
CS0LPK	67	M	C9orf72 ALS	Cedars-Sinai
CS0BUU	63	F	C9orf72 ALS	Cedars-Sinai
CS7VCZ	64	M	C9orf72 ALS	Cedars-Sinai
CS6ZLD	Unknown	F	C9orf72 ALS	Cedars-Sinai
CS8KT3	60	M	C9orf72 ALS	Cedars-Sinai

Supplementary Table 2. Demographic information for iPSC lines used in excitotoxicity experiments.

Guide RNAs	Target Transcript	Sequence (5’ – 3’)
Guide NT-1 (lenti)	N/A	GTAATGCCTGGCTTGTCGACGCATAGTCTG
Guide NT-1 (AAV)	N/A	GTAATGCCTGGCTTGTCGACGCAATAGTCTG
Guide NT-2 (lenti)	N/A	GTAATGGCCTGGCTTGTCGACGCATAGTCTG
Guide NT-2 (AAV)	N/A	GTAATGCTGGCTTGTCGACGCATAGTCTG
Guide 1	C9orf72 sense	CTTGTTCACCCTCAGCGAGTACTGTGAGAG
Guide 2	C9orf72 sense	CAGGTCTTTTCTTGTTCACCCTCAGCGAGT
Guide 3	C9orf72 sense	TAATCTTTATCAGGTCTTTTCTTGTTCACC
Guide 4	C9orf72 sense	TTCTTCTGGTTAATCTTTATCAGGTCTTTT
Guide 5	C9orf72 sense	CCTCCTTGTTTTCTTCTGGTTAATCTTTAT
Guide 6	C9orf72 sense	CGGTTGTTTCCCTCCTTGTTTTCTTCTGGT
Guide 7	C9orf72 sense	CTACAGGCTGCGGTTGTTTCCCTCCTTGTT
Guide 8	C9orf72 sense	CCAGAGCTTGCTACAGGCTGCGGTTGTTTC
Guide 9	C9orf72 sense	CTCCTGAGTTCCAGAGCTTGCTACAGGCTG
Guide 10	C9orf72 sense	TAGCGCGCGACTCCTGAGTTCCAGAGCTTG
Guide 11	C9orf72 antisense	CGCAGGCGGTGGCGCAGTGGGTGAGTGAGGA
Guide 11 (lenti)	C9orf72 antisense	CGCAGGCGGTGGCGAGTGGGTG
Guide 12	C9orf72 antisense	TGCGCCCGCGGCGGCGGAGGCGCAGGCGGT
Guide 13	C9orf72 antisense	TTAACTTTCCCTCTCATTTCTCTGACCGAA
Guide 14	C9orf72 antisense	TTCGGCTGCCGGGAAGAGGCGCGGGTAGAA
Guide 17	C9orf72 antisense	TCCCTCTCATTTCTCTGACCGAAGCTGGGT
Guide 17 (lenti)	C9orf72 antisense	TCCCTCTCATTTCTCTGACCGAA
Zebrafish guide 1	G_4C_2 zebrafish	AAACAAATTCAAAGTAAGATTAGCAAGCTT
Zebrafish guide 2	G_4C_2 zebrafish	AGGATCCCTGTAAACAAATTCAAAGTAAGA
Zebrafish guide 3	C_4G_2 zebrafish	ACTAGTGGATCCGAGCTCGGTACCAAGCTT
Zebrafish guide 4	C_4G_2 zebrafish	CTGGACTAGTGGATCCGAGCTCGGTACCAA

Supplementary Table 3. gRNA sequences.

Antigen	Species	Dilution	Origin
GABAA Receptor (α1 subunit)	Rabbit	1:100	Sigma (G4416)
GluR2	Mouse	1:100	Invitrogen (32-0300)
Map2	Mouse	1:500	Millipore (MAB3418)
TuJ1	Rabbit	1:500	Cell Signalling (5568T)
Anti-mouse Alexa Fluor 488	Goat	1:1000	Invitrogen (A21131)
Anti-rabbit Alexa Fluor 488	Donkey	1:1000	BioLegend (406416)
Anti-rabbit Alexa Fluor 546	Goat	1:1000	Invitrogen (A11035)

Supplementary Table 4. Antibodies used for immunocytochemistry.

**Primers**	**Source**	**Sequence (5' – 3')**
C9 intron 1a Fwd	Sigma	CCCCACTACTTGCTCTCACA
C9 intron 1a Rev	Sigma	CGGTTGTTTCCCTCCTTGTT
C9 V2 Exon 1b+2 Fwd	Sigma	GCGGTGGCGAGTGGATAT
C9 V2 Exon 1b+2 Rev	Sigma	TGGGCAAAGAGTCGACATCA
C9 V2 Exon 1b+2 Rev MGB probe	Sigma	ATTTGGATAATGTGACAGTTGG
C9 antisense Fwd	Sigma	AGTCGCTAGAGGCGAAAGC
C9 antisense Rev	Sigma	CGAGTGGGTGAGTGAGGAG
CasRx Fwd	Sigma	CCTCGCCGAATACATTACCAACGCC
CasRx Rev	Sigma	ATTGTTGAAAGCGGCCCTATGGTGC
GAPDH (human) Fwd	Sigma	GTCTCCTCTGACTTCAACAGCG
GAPDH (human) Rev	Sigma	ACCACCCTGTTGCTGTAGCCAA
OCT4 Fwd	Sigma	ATGCATTCAAACTGAGGTGCCTGC
OCT4 Rev	Sigma	AACTTCACCTTCCCTCGAACGAGT
TUJ1 Fwd	Sigma	CCCGGAACCATGGACAGTGT
TUJ1 Rev	Sigma	TGACCCTTGGCCCAGTTGTT
MAP2 Fwd	Sigma	GGATATGCGCTGATTCTTCA
MAP2 Rev	Sigma	CTTTCCGTTCATCTGCCATT
Gapdh (mouse) Fwd/Rev mix	IDT	Predesigned catalog (Mm.PT.39a.1)
STMN2 Fwd	IDT	AGCTGTCCATGCTGTCACTG
STMN2 Rev	IDT	GGTGGCTTCAAGATCAGCTC
Truncated STMN2 Fwd	IDT	GGACTCGGCAGAAGACCTTC
Truncated STMN2 Rev	IDT	GCAGGCTGTCTGTCTCTCTC
HDGFL2 CE Fwd	IDT	TCACACCTGAGAAGAAAGCAG
HDGFL2 CE Rev	IDT	TCCTCTCTTCTGTGTCCCTCT

Supplementary Table 5. List of qPCR primers.

Uncropped Western blot from Supplemental Figure 6m.

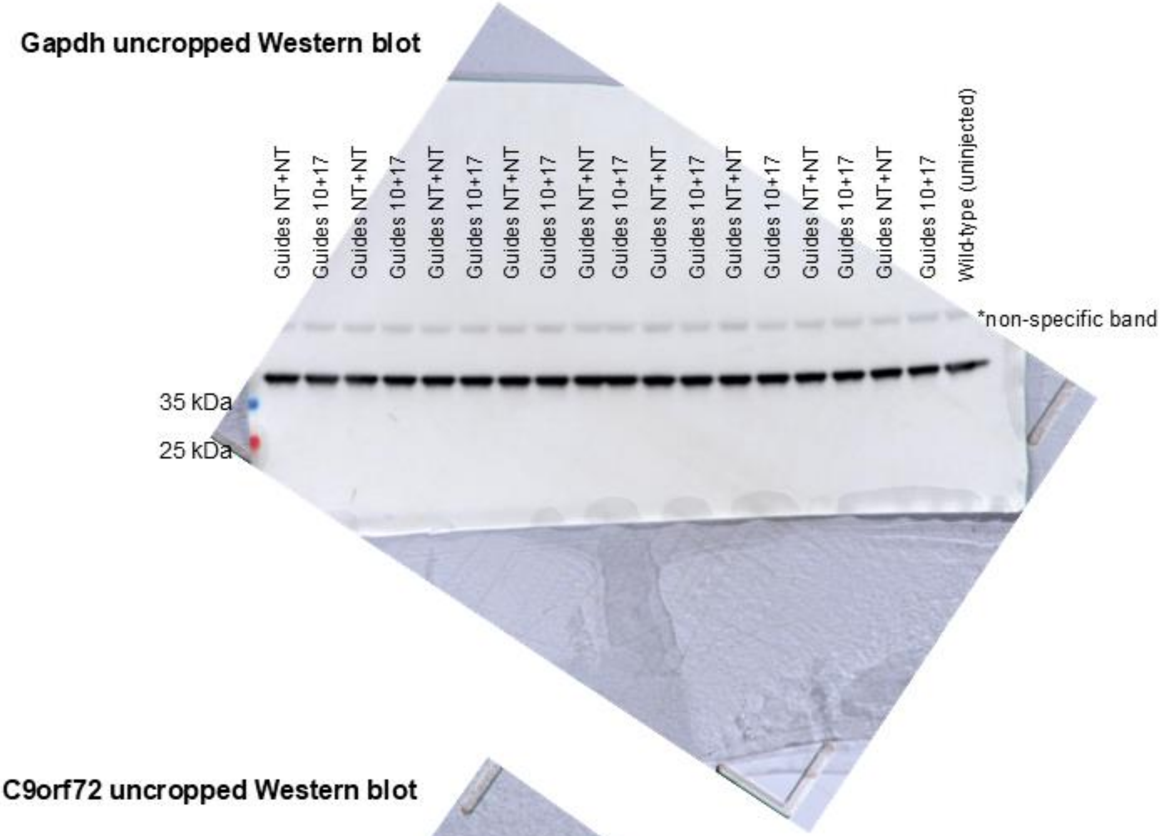

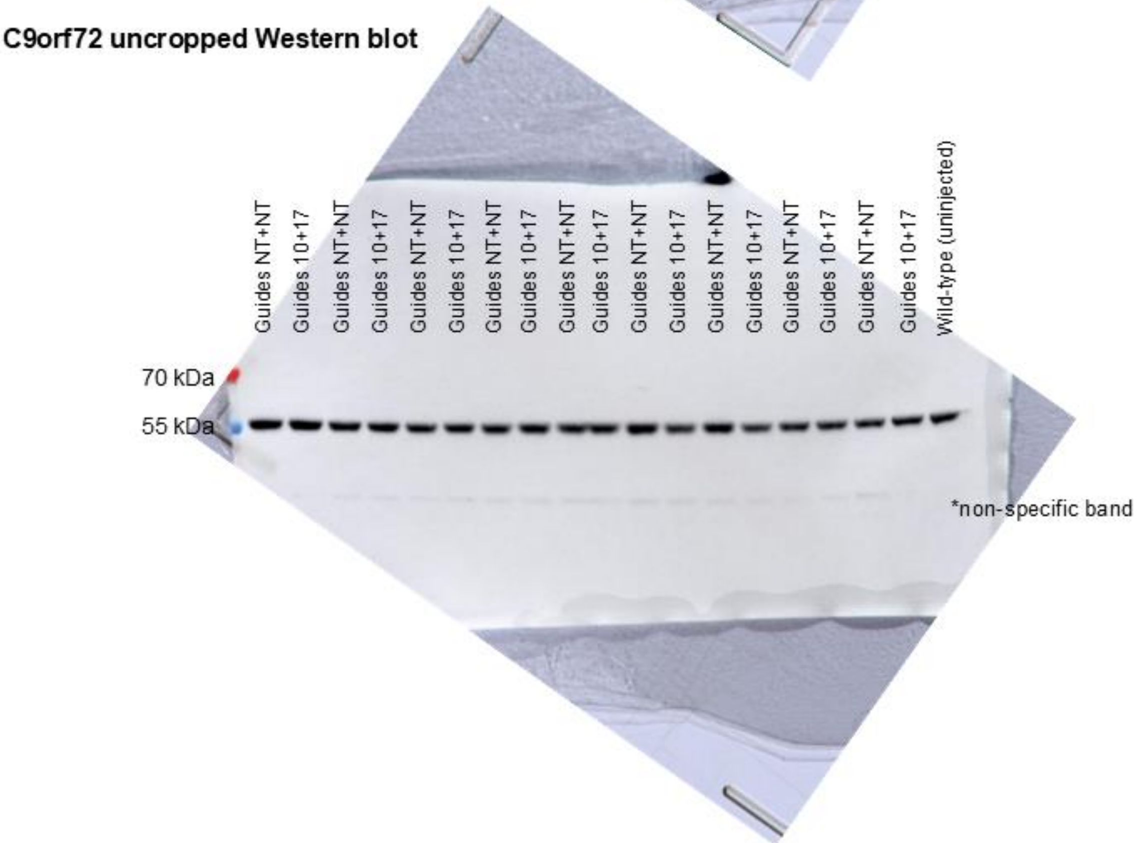

Supplement: Supplementary file 1 — Supplementary Information [file 41467_2024_55550_MOESM1_ESM.pdf]
