# Supplementary material for: Dual-targeting CRISPR-CasRx reduces C9orf72 ALS/FTD sense and antisense repeat RNAs in vitro and in vivo
Source: Nat Commun. 2025 Jan 8;16:459. doi: 10.1038/s41467-024-55550-x (PMC11711508; doi:10.1038/s41467-024-55550-x)
Supplement: Supplementary file 2 — Description of Additional Supplementary Files [file 41467_2024_55550_MOESM2_ESM.pdf]

Description of Additional Supplementary Files

File Name: Supplementary Data 1
Description: i3neuron RNA-seq summary data
