# Supplementary material for: Dual-targeting CRISPR-CasRx reduces C9orf72 ALS/FTD sense and antisense repeat RNAs in vitro and in vivo
Source: Nat Commun. 2025 Jan 8;16:459. doi: 10.1038/s41467-024-55550-x (PMC11711508; doi:10.1038/s41467-024-55550-x)
Supplement: Supplementary file 4 — Reporting Summary [file 41467_2024_55550_MOESM4_ESM.pdf]

nature portfolio

Corresponding author(s): Isaacs, Adrian M

Last updated by author(s): Nov 14, 2024

Reporting Summary

Nature Portfolio wishes to improve the reproducibility of the work that we publish. This form provides structure for consistency and transparency in reporting. For further information on Nature Portfolio policies, see our Editorial Policies and the Editorial Policy Checklist.

Statistics

For all statistical analyses, confirm that the following items are present in the figure legend, table legend, main text, or Methods section.

n/a	Confirmed	
☐	☒	The exact sample size (*n*) for each experimental group/condition, given as a discrete number and unit of measurement
☐	☒	A statement on whether measurements were taken from distinct samples or whether the same sample was measured repeatedly
☐	☒	The statistical test(s) used AND whether they are one- or two-sided<br>*Only common tests should be described solely by name; describe more complex techniques in the Methods section.*
☒	☐	A description of all covariates tested
☐	☒	A description of any assumptions or corrections, such as tests of normality and adjustment for multiple comparisons
☐	☒	A full description of the statistical parameters including central tendency (e.g. means) or other basic estimates (e.g. regression coefficient) AND variation (e.g. standard deviation) or associated estimates of uncertainty (e.g. confidence intervals)
☐	☒	For null hypothesis testing, the test statistic (e.g. *F*, *t*, *r*) with confidence intervals, effect sizes, degrees of freedom and *P* value noted<br>*Give P values as exact values whenever suitable.*
☒	☐	For Bayesian analysis, information on the choice of priors and Markov chain Monte Carlo settings
☒	☐	For hierarchical and complex designs, identification of the appropriate level for tests and full reporting of outcomes
☒	☐	Estimates of effect sizes (e.g. Cohen's *d*, Pearson's *r*), indicating how they were calculated

Our web collection on statistics for biologists contains articles on many of the points above.

Software and code

Policy information about availability of computer code

Data collection	No custom code used, just standard analysis software: video-tracking software (ZebraLab, Viewpoint Behavior Technology)
Data analysis	No custom code used, just standard analysis software: GraphPad Prism for statistical analysis. For RNA-seq DEseq2 package (v1.36.0), R packages EnhancedVolcano (v.1.14.0), ggplot2 (v.3.4.1), and ggsci (v.2.9) were used for data visualisation

For manuscripts utilizing custom algorithms or software that are central to the research but not yet described in published literature, software must be made available to editors and reviewers. We strongly encourage code deposition in a community repository (e.g. GitHub). See the Nature Portfolio guidelines for submitting code & software for further information.

Data

Policy information about availability of data

All manuscripts must include a data availability statement. This statement should provide the following information, where applicable:

- Accession codes, unique identifiers, or web links for publicly available datasets
- A description of any restrictions on data availability
- For clinical datasets or third party data, please ensure that the statement adheres to our policy

All data are available in the main text or the supplementary materials, or in the source data files provided. RNA-seq data are deposited on GEO accession number: GSE255022.

Research involving human participants, their data, or biological material

Policy information about studies with human participants or human data. See also policy information about sex, gender (identity/presentation), and sexual orientation and race, ethnicity and racism.

Reporting on sex and gender	N/A
Reporting on race, ethnicity, or other socially relevant groupings	N/A
Population characteristics	N/A
Recruitment	N/A
Ethics oversight	N/A

Note that full information on the approval of the study protocol must also be provided in the manuscript.

Field-specific reporting

Please select the one below that is the best fit for your research. If you are not sure, read the appropriate sections before making your selection.

☒ Life sciences ☐ Behavioural & social sciences ☐ Ecological, evolutionary & environmental sciences

For a reference copy of the document with all sections, see nature.com/documents/nr-reporting-summary-flat.pdf

Life sciences study design

All studies must disclose on these points even when the disclosure is negative.

Sample size	For iPSC-neuron experiments, 3 independent C9orf72 lines were used. For mouse experiments, a minimum of 7 animals were included per group, which was determined by the amount of AAV we were able to produce.
Data exclusions	No data excluded
Replication	All iPSC-neuron experiments were done 3 independent times, unless otherwise noted in figure legend/methods. All replicates showed the same trends.
Randomization	Mice were randomly assigned into experimental groups.
Blinding	Experimenters were blind to genotype when conducting P0 ICV AAV injections and then unblinded thereafter.

Reporting for specific materials, systems and methods

We require information from authors about some types of materials, experimental systems and methods used in many studies. Here, indicate whether each material, system or method listed is relevant to your study. If you are not sure if a list item applies to your research, read the appropriate section before selecting a response.

Materials & experimental systems

n/a	Involved in the study	
☐	☒	Antibodies
☐	☒	Eukaryotic cell lines
☒	☐	Palaeontology and archaeology
☐	☒	Animals and other organisms
☒	☐	Clinical data
☒	☐	Dual use research of concern
☒	☐	Plants

Methods

n/a	Involved in the study	
☒	☐	ChIP-seq
☒	☐	Flow cytometry
☒	☐	MRI-based neuroimaging

Antibodies

Antibodies used	ICC: GABAA Receptor α 1 subunit (Sigma G4416, 1:100), GluR2 (Invitrogen 32-0300, 1:100), Map2 (Millipore MAB3418, 1:500), Tuj1 (Cell Signaling 5568T, 1:500), AlexaFluor647-conjugated HA (Biolegend 16B12, 1:1000)<br>IHC: GFAP (Abcam AB5804, 1:500), Iba1 (FUJIFILM 019-19741, 1:500), RFP (VWR ROCK600-401-379, 1:1000), NeuN (Sigma ABN91, 1:500), GFAP (Invitrogen 13-0300, 1:500)<br>WB: C9orf72 (GeneTexGTX634482, 1:1000), Gapdh (Cell Signaling 14C10, 1:1000)
Validation	Primary antibodies validated by manufacturers using immunoblotting and immunostaining.

Eukaryotic cell lines

Policy information about cell lines and Sex and Gender in Research

Cell line source(s)	C9orf72 patient iPSC lines were kindly provided by Prof Siddharthan Chandran and Dr Bhuvaneish Selvaraj, University of Edinburgh and have been previously described (Selvaraj et al. 2018). Answer ALS iPSC lines provided by Cedars Sinai. HEK293T cells were from ATCC.
Authentication	Lines previously described (Selvaraj et al. 2018), or authenticated by repository (Cedars Sinai). The HEK293T cells were not re-authenticated in our laboratory.
Mycoplasma contamination	All lines were regularly tested for mycoplasma contamination throughout the study.
Commonly misidentified lines<br>(See ICLAC register)	N/A

Animals and other research organisms

Policy information about studies involving animals; ARRIVE guidelines recommended for reporting animal research, and Sex and Gender in Research

Laboratory animals	For 149R AAV experiments, all animals were wild-type C57bl6/J acquired from either Envigo or Charles River. C9orf72 BAC mice were acquired from Jackson Laboratories and are of strain FVB/NJ-Tg(C9orf72)500Lpwr/J (Stock No: 029099). All mice were treated at P0.
Wild animals	N/A
Reporting on sex	All animals, male and female, were included in these studies in Mendelian ratios.
Field-collected samples	N/A
Ethics oversight	Animals were maintained and experimental procedures performed in accordance with the UK Animal Scientific Procedure Act 1986, under project and personal licenses issued by the UK Home Office, and approved by the UCL and the Institute of Prion Diseases Animal Welfare and Ethical Review Bodies.

Note that full information on the approval of the study protocol must also be provided in the manuscript.

Plants

Seed stocks	N/A
Novel plant genotypes	N/A
Authentication	N/A
